# American Medical Association

**Published:** 1866-04

**Authors:** William B. Atkinson

**Affiliations:** Permanent Secretary, Philadelphia


*American Medical Association.*—The Seventeenth Annual Session of this Association will be held in the city of Baltimore, May 1st, 1866. The following Committees were appointed to report at that time:

On Prize Essays, Dr. Austin Flint, Sr., New York, Chairman.

On Quarantine, Dr. Wilson Jewell, Pa., Chairman.

On so-called Spotted Fever, Dr. Jas. J. Levick, Pa., Chairman.

On Ligature of the Subclavian Artery, Dr. William Parker, N. Y., Chairman.

On Tracheotomy in Membranous Croup, Dr. Alex. N. Dougherty, N. J., Chairman.

On Rank of Medical Corps in the Army, Dr. C. S. Tripler, U. S. A., Chairman.

On Rank of Medical Corps in the Navy, Dr. T. L. Smith, N. Y., Chairman.

On Medical Literature, Dr. C. A. Lee, N. Y., Chairman.

On Medical Education, Dr. Samuel D. Gross, Pa., Chairman.

On American Necrology, Dr. C. C. Cox, Md., Chairman.

On Patent Rights and Medical Men, Dr. David Prince, Illinois, Chairman.

On Alcohol and its Relations to Man, Dr. Gerard E. Morgan, Md., Chairman.

On Insanity, Dr. Alfred Hitchcock, Mass., Chairman.

On Milk Sickness, Dr. Robert Thompson, Ohio, Chairman.

On the relation which the Doctrine of the Correlation and Conservation of Forces bears to the Physiological and Pathological Condition of the Human System, Dr. S. L. Loomis, D. C., Chairman.

On the Progress of Medical Science, Dr. Jerome Candee Smith, N. Y., Chairman.

On Diphtheria, Dr. H. D. Holton, Vt., Chairman.

On the Comparative Value of Life in City and Country, Dr. Edw. Jarvis, Mass., Chairman.

On Drainage and Sewerage of Cities in their Influence on Health, Dr. Wilson Jewell, Pa., Chairman.

What Effects has Civilization on the Duration of Human Life, Dr. Augustus A. Gould, Mass., Chairman.

On Disinfectants, Dr. E. M. Hunt, N. J. Chairman.

On Compulsory Vaccination, Dr. A. Nelson Bell, N. Y., Chairman.

On Strangulated Hernia, Dr. W. F. Peck, Iowa, Chairman.

On the Causes and Pathology of Pyæmia, Dr. J. J. Woodward, U. S. A., Chairman.

On the Use of Plaster Paris in Surgery, Dr. Jas. L. Little, N. Y., Chairman.

On the Etiological and Pathological Relations of Epidemic Erysipelas, Spotted Fever, Diphtheria, and Scarlatina, Dr. N S. Davis, Ill., Chairman.

On Meteorology, Medical Topography, and Epidemics:

| | |
|---|---|
| Dr. J. C. Weston, Me. | Dr. D. Francis Condie, Pa. |
| " P. A. Stackpole, N. H. | " T. Antisell, D. C. |
| " C. L. Allen, Vt. | " O. S. Mahon, Md. |
| " A. C. Garratt, Mass. | " T. M. Logan, Cal. |
| " C. W. Parsons, R. I. | " R. C. Hamill, Ill. |
| " B. H. Catlin, Conn. | " J. W. H. Baker, Iowa. |
| " E. M. Chapman, N. Y. | " Abm. Sager, Mich. |
| " E. M. Hunt, N. J. | " J. W. Russell, Ohio. |

WILLIAM B. ATKINSON,
*Permanent Secretary, Philadelphia.*

